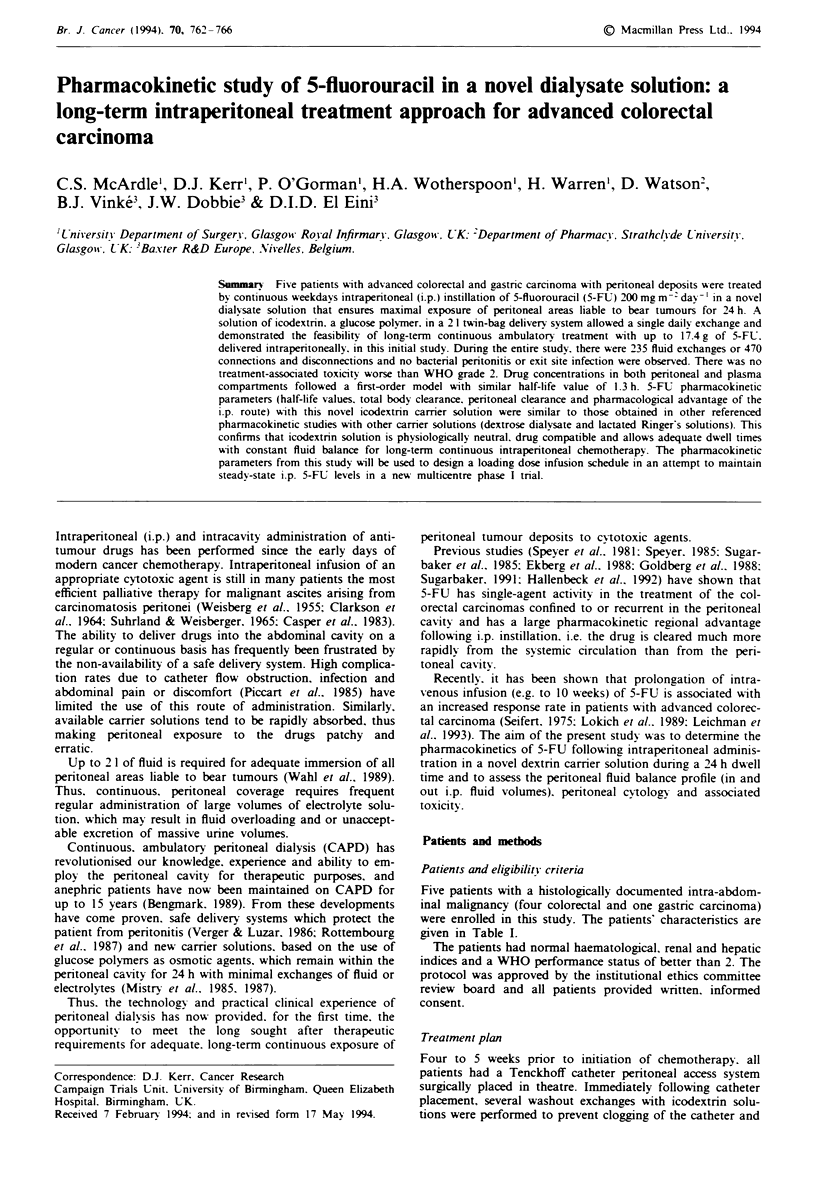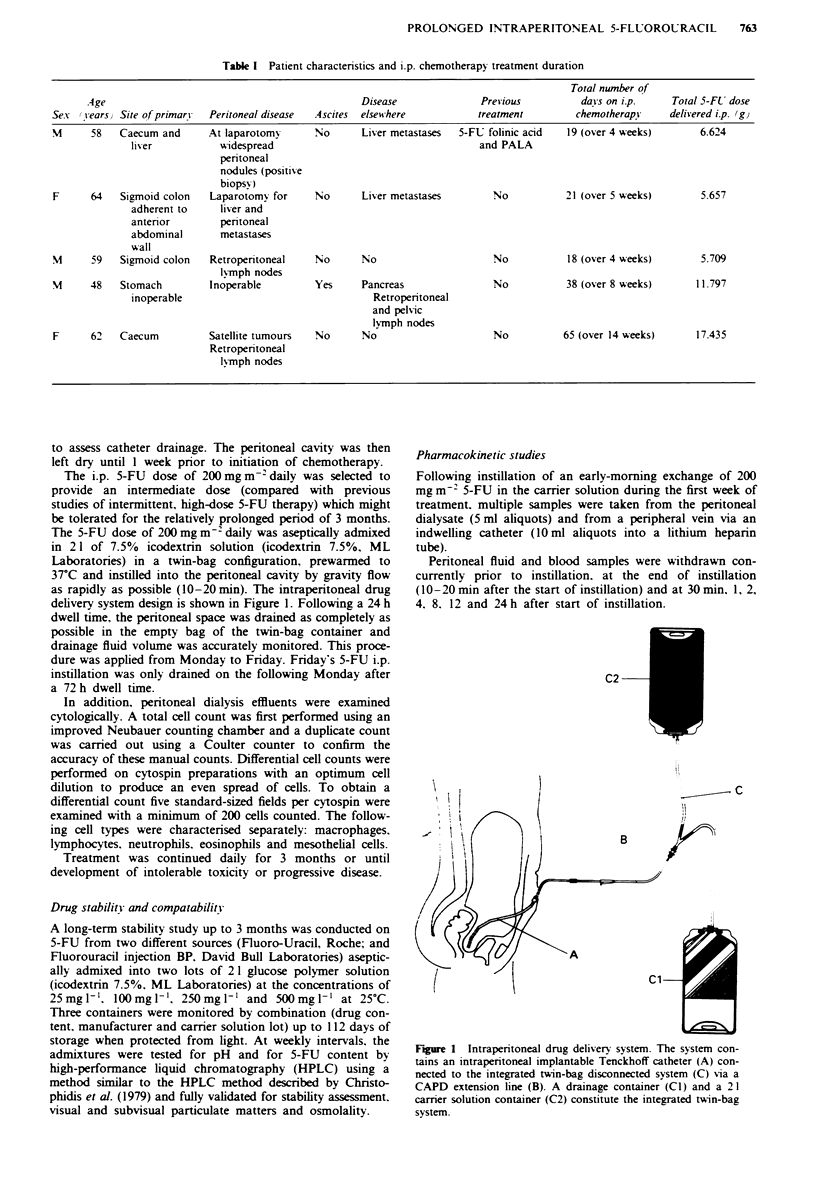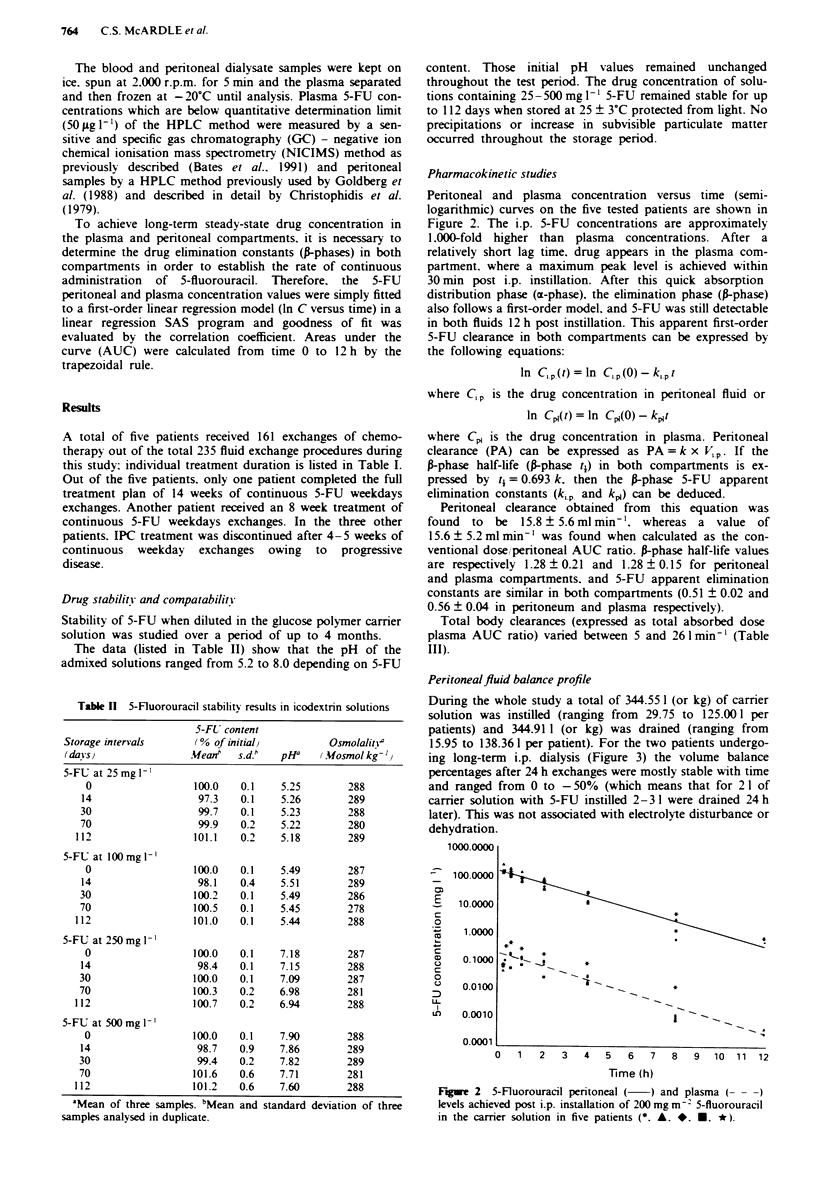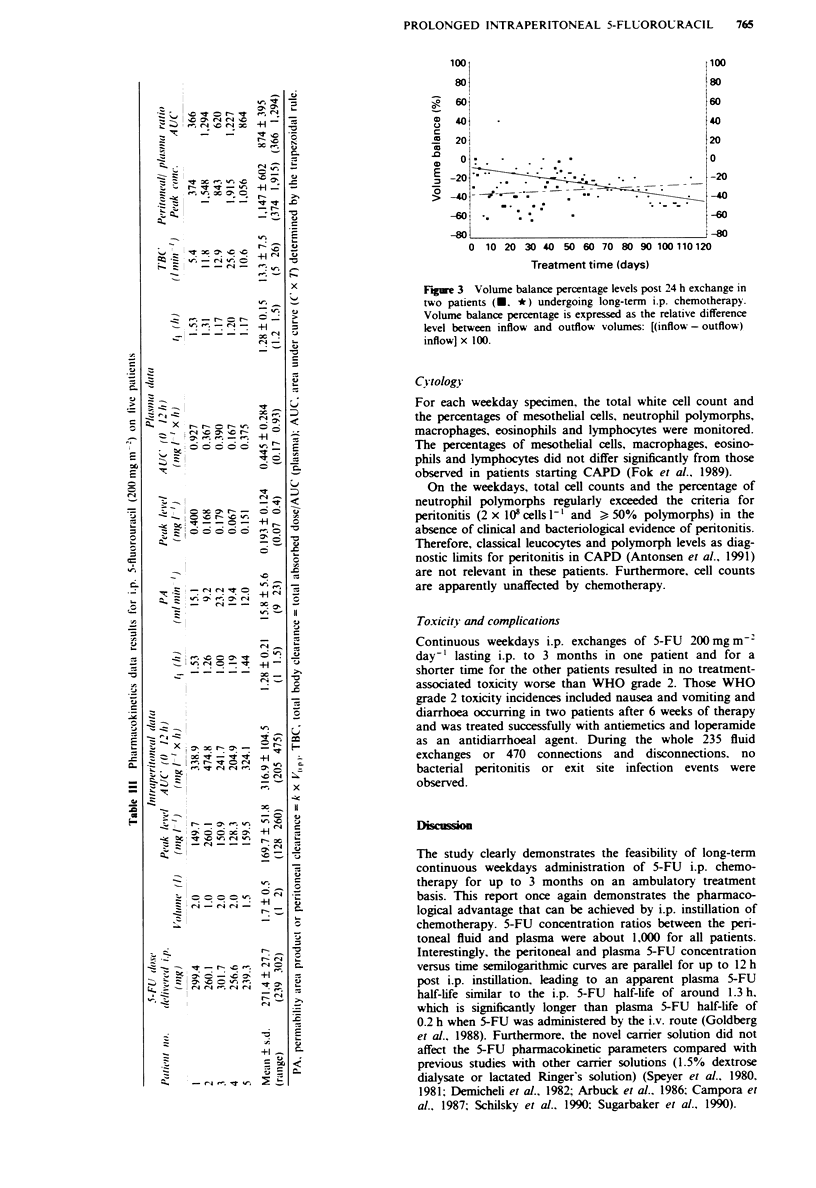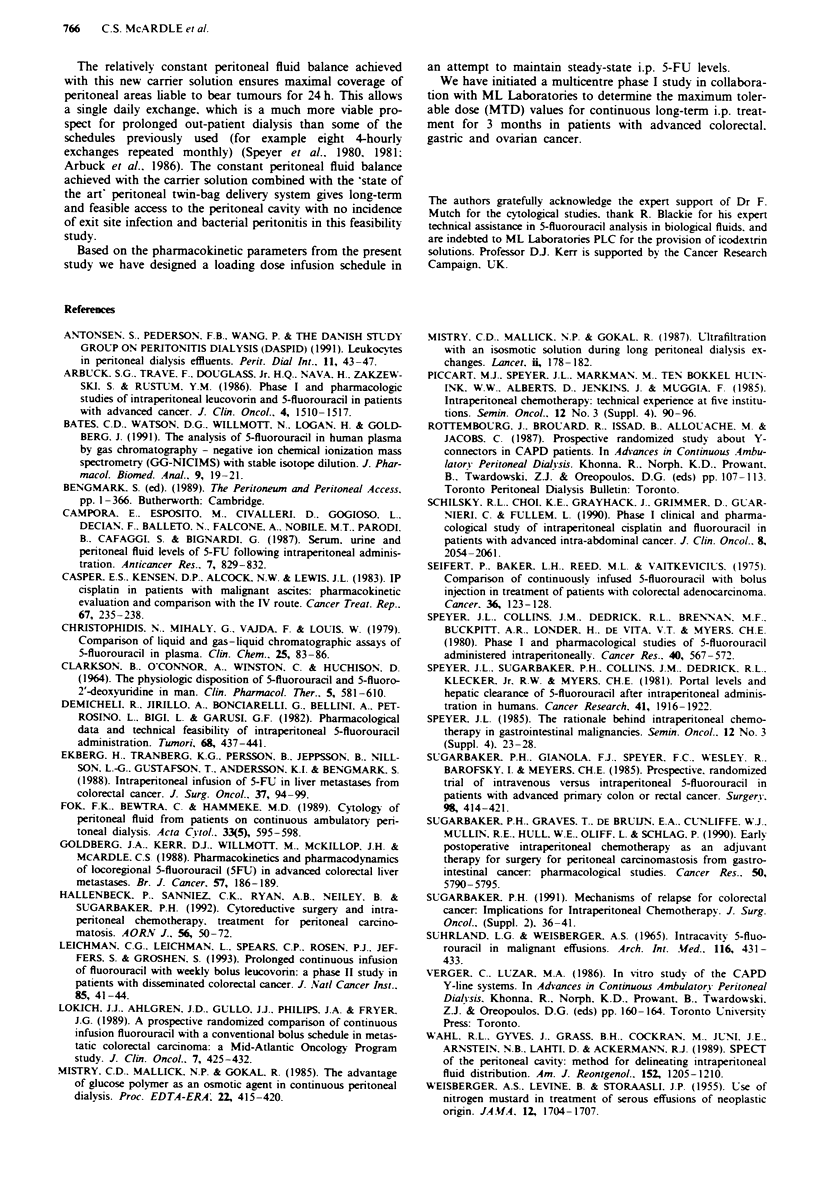# Pharmacokinetic study of 5-fluorouracil in a novel dialysate solution: a long-term intraperitoneal treatment approach for advanced colorectal carcinoma.

**DOI:** 10.1038/bjc.1994.392

**Published:** 1994-10

**Authors:** C. S. McArdle, D. J. Kerr, P. O'Gorman, H. A. Wotherspoon, H. Warren, D. Watson, B. J. Vinké, J. W. Dobbie, D. I. el Eini

**Affiliations:** University Department of Surgery, Glasgow Royal Infirmary, UK.

## Abstract

Five patients with advanced colorectal and gastric carcinoma with peritoneal deposits were treated by continuous weekdays intraperitoneal (i.p.) instillation of 5-fluorouracil (5-FU) 200 mg m-2 day-1 in a novel dialysate solution that ensures maximal exposure of peritoneal areas liable to bear tumours for 24 h. A solution of icodextrin, a glucose polymer, in a 21 twin-bag delivery system allowed a single daily exchange and demonstrated the feasibility of long-term continuous ambulatory treatment with up to 17.4 g of 5-FU, delivered intraperitoneally, in this initial study. During the entire study, there were 235 fluid exchanges or 470 connections and disconnections and no bacterial peritonitis or exit site infection were observed. There was no treatment-associated toxicity worse than WHO grade 2. Drug concentrations in both peritoneal and plasma compartments followed a first-order model with similar half-life value of 1.3 h. 5-FU pharmacokinetic parameters (half-life values, total body clearance, peritoneal clearance and pharmacological advantage of the i.p. route) with this novel icodextrin carrier solution were similar to those obtained in other referenced pharmacokinetic studies with other carrier solutions (dextrose dialysate and lactated Ringer's solutions). This confirms that icodextrin solution is physiologically neutral, drug compatible and allows adequate dwell times with constant fluid balance for long-term continuous intraperitoneal chemotherapy. The pharmacokinetic parameters from this study will be used to design a loading dose infusion schedule in an attempt to maintain steady-state i.p. 5-FU levels in a new multicentre phase I trial.


Br. J. Cancer (1994), 70, 762-766 © Macmillan Press Ltd., 1994

### Pharmacokinetic study of 5-fluorouracil in a novel dialysate solution: a long-term intraperitoneal treatment approach for advanced colorectal carcinoma

C.S. McArdle[1], D.J. Kerr[1], P. O'Gorman[1], H.A. Wotherspoon[1], H. Warren[1], D. Watson[2], B.J. Vinké[3], J.W. Dobbie[3] & D.I.D. El Eini[3]

[1]*University Department of Surgery, Glasgow Royal Infirmary, Glasgow, UK;* [2]*Department of Pharmacy, Strathclyde University, Glasgow, UK;* [3]*Baxter R&D Europe, Nivelles, Belgium.*

**Summary** Five patients with advanced colorectal and gastric carcinoma with peritoneal deposits were treated by continuous weekdays intraperitoneal (i.p.) instillation of 5-fluorouracil (5-FU) 200 mg $m^{-2}$ $day^{-1}$ in a novel dialysate solution that ensures maximal exposure of peritoneal areas liable to bear tumours for 24 h. A solution of icodextrin, a glucose polymer, in a 2 l twin-bag delivery system allowed a single daily exchange and demonstrated the feasibility of long-term continuous ambulatory treatment with up to 17.4 g of 5-FU, delivered intraperitoneally, in this initial study. During the entire study, there were 235 fluid exchanges or 470 connections and disconnections and no bacterial peritonitis or exit site infection were observed. There was no treatment-associated toxicity worse than WHO grade 2. Drug concentrations in both peritoneal and plasma compartments followed a first-order model with similar half-life value of 1.3 h. 5-FU pharmacokinetic parameters (half-life values, total body clearance, peritoneal clearance and pharmacological advantage of the i.p. route) with this novel icodextrin carrier solution were similar to those obtained in other referenced pharmacokinetic studies with other carrier solutions (dextrose dialysate and lactated Ringer's solutions). This confirms that icodextrin solution is physiologically neutral, drug compatible and allows adequate dwell times with constant fluid balance for long-term continuous intraperitoneal chemotherapy. The pharmacokinetic parameters from this study will be used to design a loading dose infusion schedule in an attempt to maintain steady-state i.p. 5-FU levels in a new multicentre phase I trial.

Intraperitoneal (i.p.) and intracavity administration of anti-tumour drugs has been performed since the early days of modern cancer chemotherapy. Intraperitoneal infusion of an appropriate cytotoxic agent is still in many patients the most efficient palliative therapy for malignant ascites arising from carcinomatosis peritonei (Weisberg *et al.*, 1955; Clarkson *et al.*, 1964; Suhrland & Weisberger, 1965; Casper *et al.*, 1983). The ability to deliver drugs into the abdominal cavity on a regular or continuous basis has frequently been frustrated by the non-availability of a safe delivery system. High complication rates due to catheter flow obstruction, infection and abdominal pain or discomfort (Piccart *et al.*, 1985) have limited the use of this route of administration. Similarly, available carrier solutions tend to be rapidly absorbed, thus making peritoneal exposure to the drugs patchy and erratic.

Up to 2 l of fluid is required for adequate immersion of all peritoneal areas liable to bear tumours (Wahl *et al.*, 1989). Thus, continuous, peritoneal coverage requires frequent regular administration of large volumes of electrolyte solution, which may result in fluid overloading and or unacceptable excretion of massive urine volumes.

Continuous, ambulatory peritoneal dialysis (CAPD) has revolutionised our knowledge, experience and ability to employ the peritoneal cavity for therapeutic purposes, and anephric patients have now been maintained on CAPD for up to 15 years (Bengmark, 1989). From these developments have come proven, safe delivery systems which protect the patient from peritonitis (Verger & Luzar, 1986; Rottembourg *et al.*, 1987) and new carrier solutions, based on the use of glucose polymers as osmotic agents, which remain within the peritoneal cavity for 24 h with minimal exchanges of fluid or electrolytes (Mistry *et al.*, 1985, 1987).

Thus, the technology and practical clinical experience of peritoneal dialysis has now provided, for the first time, the opportunity to meet the long sought after therapeutic requirements for adequate, long-term continuous exposure of peritoneal tumour deposits to cytotoxic agents.

Previous studies (Speyer *et al.*, 1981; Speyer, 1985; Sugarbaker *et al.*, 1985; Ekberg *et al.*, 1988; Goldberg *et al.*, 1988; Sugarbaker, 1991; Hallenbeck *et al.*, 1992) have shown that 5-FU has single-agent activity in the treatment of the colorectal carcinomas confined to or recurrent in the peritoneal cavity and has a large pharmacokinetic regional advantage following i.p. instillation, i.e. the drug is cleared much more rapidly from the systemic circulation than from the peritoneal cavity.

Recently, it has been shown that prolongation of intravenous infusion (e.g. to 10 weeks) of 5-FU is associated with an increased response rate in patients with advanced colorectal carcinoma (Seifert, 1975; Lokich *et al.*, 1989; Leichman *et al.*, 1993). The aim of the present study was to determine the pharmacokinetics of 5-FU following intraperitoneal administration in a novel dextrin carrier solution during a 24 h dwell time and to assess the peritoneal fluid balance profile (in and out i.p. fluid volumes), peritoneal cytology and associated toxicity.

### Patients and methods

### *Patients and eligibility criteria*

Five patients with a histologically documented intra-abdominal malignancy (four colorectal and one gastric carcinoma) were enrolled in this study. The patients' characteristics are given in Table I.

The patients had normal haematological, renal and hepatic indices and a WHO performance status of better than 2. The protocol was approved by the institutional ethics committee review board and all patients provided written, informed consent.

### *Treatment plan*

Four to 5 weeks prior to initiation of chemotherapy, all patients had a Tenckhoff catheter peritoneal access system surgically placed in theatre. Immediately following catheter placement, several washout exchanges with icodextrin solutions were performed to prevent clogging of the catheter and

Correspondence: D.J. Kerr, Cancer Research Campaign Trials Unit, University of Birmingham, Queen Elizabeth Hospital, Birmingham, UK.
Received 7 February 1994; and in revised form 17 May 1994.

**Table I** Patient characteristics and i.p. chemotherapy treatment duration

| *Sex* | *Age (years)* | *Site of primary* | *Peritoneal disease* | *Ascites* | *Disease elsewhere* | *Previous treatment* | *Total number of days on i.p. chemotherapy* | *Total 5-FU dose delivered i.p. (g)* |
|---|---|---|---|---|---|---|---|---|
| M | 58 | Caecum and liver | At laparotomy widespread peritoneal nodules (positive biopsy) | No | Liver metastases | 5-FU folinic acid and PALA | 19 (over 4 weeks) | 6.624 |
| F | 64 | Sigmoid colon adherent to anterior abdominal wall | Laparotomy for liver and peritoneal metastases | No | Liver metastases | No | 21 (over 5 weeks) | 5.657 |
| M | 59 | Sigmoid colon | Retroperitoneal lymph nodes | No | No | No | 18 (over 4 weeks) | 5.709 |
| M | 48 | Stomach inoperable | Inoperable | Yes | Pancreas Retroperitoneal and pelvic lymph nodes | No | 38 (over 8 weeks) | 11.797 |
| F | 62 | Caecum | Satellite tumours Retroperitoneal lymph nodes | No | No | No | 65 (over 14 weeks) | 17.435 |

to assess catheter drainage. The peritoneal cavity was then left dry until 1 week prior to initiation of chemotherapy.

The i.p. 5-FU dose of 200 mg $m^{-2}$ daily was selected to provide an intermediate dose (compared with previous studies of intermittent, high-dose 5-FU therapy) which might be tolerated for the relatively prolonged period of 3 months. The 5-FU dose of 200 mg $m^{-2}$ daily was aseptically admixed in 2 l of 7.5% icodextrin solution (icodextrin 7.5%, ML Laboratories) in a twin-bag configuration, prewarmed to 37°C and instilled into the peritoneal cavity by gravity flow as rapidly as possible (10–20 min). The intraperitoneal drug delivery system design is shown in Figure 1. Following a 24 h dwell time, the peritoneal space was drained as completely as possible in the empty bag of the twin-bag container and drainage fluid volume was accurately monitored. This procedure was applied from Monday to Friday. Friday's 5-FU i.p. instillation was only drained on the following Monday after a 72 h dwell time.

In addition, peritoneal dialysis effluents were examined cytologically. A total cell count was first performed using an improved Neubauer counting chamber and a duplicate count was carried out using a Coulter counter to confirm the accuracy of these manual counts. Differential cell counts were performed on cytospin preparations with an optimum cell dilution to produce an even spread of cells. To obtain a differential count five standard-sized fields per cytospin were examined with a minimum of 200 cells counted. The following cell types were characterised separately: macrophages, lymphocytes, neutrophils, eosinophils and mesothelial cells.

Treatment was continued daily for 3 months or until development of intolerable toxicity or progressive disease.

*Drug stability and compatability*

A long-term stability study up to 3 months was conducted on 5-FU from two different sources (Fluoro-Uracil, Roche; and Fluorouracil injection BP, David Bull Laboratories) aseptically admixed into two lots of 2 l glucose polymer solution (icodextrin 7.5%, ML Laboratories) at the concentrations of 25 mg $l^{-1}$, 100 mg $l^{-1}$, 250 mg $l^{-1}$ and 500 mg $l^{-1}$ at 25°C. Three containers were monitored by combination (drug content, manufacturer and carrier solution lot) up to 112 days of storage when protected from light. At weekly intervals, the admixtures were tested for pH and for 5-FU content by high-performance liquid chromatography (HPLC) using a method similar to the HPLC method described by Christophidis *et al.* (1979) and fully validated for stability assessment, visual and subvisual particulate matters and osmolality.

*Pharmacokinetic studies*

Following instillation of an early-morning exchange of 200 mg $m^{-2}$ 5-FU in the carrier solution during the first week of treatment, multiple samples were taken from the peritoneal dialysate (5 ml aliquots) and from a peripheral vein via an indwelling catheter (10 ml aliquots into a lithium heparin tube).

Peritoneal fluid and blood samples were withdrawn concurrently prior to instillation, at the end of instillation (10–20 min after the start of instillation) and at 30 min, 1, 2, 4, 8, 12 and 24 h after start of instillation.

**Figure 1** Intraperitoneal drug delivery system. The system contains an intraperitoneal implantable Tenckhoff catheter (A) connected to the integrated twin-bag disconnected system (C) via a CAPD extension line (B). A drainage container (C1) and a 2 l carrier solution container (C2) constitute the integrated twin-bag system.

The blood and peritoneal dialysate samples were kept on ice, spun at 2,000 r.p.m. for 5 min and the plasma separated and then frozen at $-20°C$ until analysis. Plasma 5-FU concentrations which are below quantitative determination limit ($50\,\mu g\,l^{-1}$) of the HPLC method were measured by a sensitive and specific gas chromatography (GC) – negative ion chemical ionisation mass spectrometry (NICIMS) method as previously described (Bates *et al.*, 1991) and peritoneal samples by a HPLC method previously used by Goldberg *et al.* (1988) and described in detail by Christophidis *et al.* (1979).

To achieve long-term steady-state drug concentration in the plasma and peritoneal compartments, it is necessary to determine the drug elimination constants ($\beta$-phases) in both compartments in order to establish the rate of continuous administration of 5-fluorouracil. Therefore, the 5-FU peritoneal and plasma concentration values were simply fitted to a first-order linear regression model (ln $C$ versus time) in a linear regression SAS program and goodness of fit was evaluated by the correlation coefficient. Areas under the curve (AUC) were calculated from time 0 to 12 h by the trapezoidal rule.

### Results

A total of five patients received 161 exchanges of chemotherapy out of the total 235 fluid exchange procedures during this study; individual treatment duration is listed in Table I. Out of the five patients, only one patient completed the full treatment plan of 14 weeks of continuous 5-FU weekdays exchanges. Another patient received an 8 week treatment of continuous 5-FU weekdays exchanges. In the three other patients, IPC treatment was discontinued after 4–5 weeks of continuous weekday exchanges owing to progressive disease.

### *Drug stability and compatability*

Stability of 5-FU when diluted in the glucose polymer carrier solution was studied over a period of up to 4 months.

The data (listed in Table II) show that the pH of the admixed solutions ranged from 5.2 to 8.0 depending on 5-FU content. Those initial pH values remained unchanged throughout the test period. The drug concentration of solutions containing $25-500\,mg\,l^{-1}$ 5-FU remained stable for up to 112 days when stored at $25 \pm 3°C$ protected from light. No precipitations or increase in subvisible particulate matter occurred throughout the storage period.

**Table II** 5-Fluorouracil stability results in icodextrin solutions

| *Storage intervals (days)* | *5-FU content (% of initial) Mean*[b] | *s.d.*[b] | *pH*[a] | *Osmolality*[a] *(Mosmol kg*$^{-1}$*)* |
|---|---|---|---|---|
| 5-FU at 25 mg l$^{-1}$ | | | | |
| 0 | 100.0 | 0.1 | 5.25 | 288 |
| 14 | 97.3 | 0.1 | 5.26 | 289 |
| 30 | 99.7 | 0.1 | 5.23 | 288 |
| 70 | 99.9 | 0.2 | 5.22 | 280 |
| 112 | 101.1 | 0.2 | 5.18 | 289 |
| 5-FU at 100 mg l$^{-1}$ | | | | |
| 0 | 100.0 | 0.1 | 5.49 | 287 |
| 14 | 98.1 | 0.4 | 5.51 | 289 |
| 30 | 100.2 | 0.1 | 5.49 | 286 |
| 70 | 100.5 | 0.1 | 5.45 | 278 |
| 112 | 101.0 | 0.1 | 5.44 | 288 |
| 5-FU at 250 mg l$^{-1}$ | | | | |
| 0 | 100.0 | 0.1 | 7.18 | 287 |
| 14 | 98.4 | 0.1 | 7.15 | 288 |
| 30 | 100.0 | 0.1 | 7.09 | 287 |
| 70 | 100.3 | 0.2 | 6.98 | 281 |
| 112 | 100.7 | 0.2 | 6.94 | 288 |
| 5-FU at 500 mg l$^{-1}$ | | | | |
| 0 | 100.0 | 0.1 | 7.90 | 288 |
| 14 | 98.7 | 0.9 | 7.86 | 289 |
| 30 | 99.4 | 0.2 | 7.82 | 289 |
| 70 | 101.6 | 0.6 | 7.71 | 281 |
| 112 | 101.2 | 0.6 | 7.60 | 288 |

[a]Mean of three samples. [b]Mean and standard deviation of three samples analysed in duplicate.

### *Pharmacokinetic studies*

Peritoneal and plasma concentration versus time (semi-logarithmic) curves on the five tested patients are shown in Figure 2. The i.p. 5-FU concentrations are approximately 1,000-fold higher than plasma concentrations. After a relatively short lag time, drug appears in the plasma compartment, where a maximum peak level is achieved within 30 min post i.p. instillation. After this quick absorption distribution phase ($\alpha$-phase), the elimination phase ($\beta$-phase) also follows a first-order model, and 5-FU was still detectable in both fluids 12 h post instillation. This apparent first-order 5-FU clearance in both compartments can be expressed by the following equations:

$$\ln C_{i.p.}(t) = \ln C_{i.p.}(0) - k_{i.p.}t$$

where $C_{i.p.}$ is the drug concentration in peritoneal fluid or

$$\ln C_{pl}(t) = \ln C_{pl}(0) - k_{pl}t$$

where $C_{pl}$ is the drug concentration in plasma. Peritoneal clearance (PA) can be expressed as $PA = k \times V_{i.p.}$. If the $\beta$-phase half-life ($\beta$-phase $t_{\frac{1}{2}}$) in both compartments is expressed by $t_{\frac{1}{2}} = 0.693/k$, then the $\beta$-phase 5-FU apparent elimination constants ($k_{i.p.}$ and $k_{pl}$) can be deduced.

Peritoneal clearance obtained from this equation was found to be $15.8 \pm 5.6\,ml\,min^{-1}$, whereas a value of $15.6 \pm 5.2\,ml\,min^{-1}$ was found when calculated as the conventional dose/peritoneal AUC ratio. $\beta$-phase half-life values are respectively $1.28 \pm 0.21$ and $1.28 \pm 0.15$ for peritoneal and plasma compartments, and 5-FU apparent elimination constants are similar in both compartments ($0.51 \pm 0.02$ and $0.56 \pm 0.04$ in peritoneum and plasma respectively).

Total body clearances (expressed as total absorbed dose/plasma AUC ratio) varied between 5 and $26\,l\,min^{-1}$ (Table III).

### *Peritoneal fluid balance profile*

During the whole study a total of 344.55 l (or kg) of carrier solution was instilled (ranging from 29.75 to 125.00 l per patients) and 344.91 l (or kg) was drained (ranging from 15.95 to 138.36 l per patient). For the two patients undergoing long-term i.p. dialysis (Figure 3) the volume balance percentages after 24 h exchanges were mostly stable with time and ranged from 0 to $-50\%$ (which means that for 2 l of carrier solution with 5-FU instilled 2–3 l were drained 24 h later). This was not associated with electrolyte disturbance or dehydration.

**Figure 2** 5-Fluorouracil peritoneal (——) and plasma (- - -) levels achieved post i.p. installation of $200\,mg\,m^{-2}$ 5-fluorouracil in the carrier solution in five patients (•, ▲, ◆, ■, ★).

**Table III** Pharmacokinetics data results for i.p. 5-fluorouracil (200 mg m$^{-2}$) on five patients

| Patient no. | 5-FU dose delivered i.p. (mg) | Volume (l) | Intraperitoneal data: Peak level (mg l$^{-1}$) | Intraperitoneal data: AUC (0 12 h) (mg l$^{-1}$ × h) | Intraperitoneal data: $t_{1/2}$ (h) | Intraperitoneal data: PA (ml min$^{-1}$) | Plasma data: Peak level (mg l$^{-1}$) | Plasma data: AUC (0 12 h) (mg l$^{-1}$ × h) | Plasma data: $t_{1/2}$ (h) | TBC (l min$^{-1}$) | Peritoneal/ plasma ratio: Peak conc. | Peritoneal/ plasma ratio: AUC |
|---|---|---|---|---|---|---|---|---|---|---|---|---|
| 1 | 299.4 | 2.0 | 149.7 | 338.9 | 1.53 | 15.1 | 0.400 | 0.927 | 1.53 | 5.4 | 374 | 366 |
| 2 | 260.1 | 1.0 | 260.1 | 474.8 | 1.26 | 9.2 | 0.168 | 0.367 | 1.31 | 11.8 | 1,548 | 1,294 |
| 3 | 301.7 | 2.0 | 150.9 | 241.7 | 1.00 | 23.2 | 0.179 | 0.390 | 1.17 | 12.9 | 843 | 620 |
| 4 | 256.6 | 2.0 | 128.3 | 204.9 | 1.19 | 19.4 | 0.067 | 0.167 | 1.20 | 25.6 | 1,915 | 1,227 |
| 5 | 239.3 | 1.5 | 159.5 | 324.1 | 1.44 | 12.0 | 0.151 | 0.375 | 1.17 | 10.6 | 1,056 | 864 |
| Mean ± s.d. | 271.4 ± 27.7 | 1.7 ± 0.5 | 169.7 ± 51.8 | 316.9 ± 104.5 | 1.28 ± 0.21 | 15.8 ± 5.6 | 0.193 ± 0.124 | 0.445 ± 0.284 | 1.28 ± 0.15 | 13.3 ± 7.5 | 1,147 ± 602 | 874 ± 395 |
| (range) | (239 302) | (1 2) | (128 260) | (205 475) | (1 1.5) | (9 23) | (0.07 0.4) | (0.17 0.93) | (1.2 1.5) | (5 26) | (374 1,915) | (366 1,294) |

PA, permability area product or peritoneal clearance = $k \times V_{(i.p.)}$. TBC, total body clearance = total absorbed dose/AUC (plasma); AUC, area under curve ($C \times T$) determined by the trapezoidal rule.

**Figure 3** Volume balance percentage levels post 24 h exchange in two patients (■, ★) undergoing long-term i.p. chemotherapy. Volume balance percentage is expressed as the relative difference level between inflow and outflow volumes: [(inflow − outflow) inflow] × 100.

### *Cytology*

For each weekday specimen, the total white cell count and the percentages of mesothelial cells, neutrophil polymorphs, macrophages, eosinophils and lymphocytes were monitored. The percentages of mesothelial cells, macrophages, eosinophils and lymphocytes did not differ significantly from those observed in patients starting CAPD (Fok *et al.*, 1989).

On the weekdays, total cell counts and the percentage of neutrophil polymorphs regularly exceeded the criteria for peritonitis ($2 \times 10^8$ cells l$^{-1}$ and $\geqslant 50\%$ polymorphs) in the absence of clinical and bacteriological evidence of peritonitis. Therefore, classical leucocytes and polymorph levels as diagnostic limits for peritonitis in CAPD (Antonsen *et al.*, 1991) are not relevant in these patients. Furthermore, cell counts are apparently unaffected by chemotherapy.

### *Toxicity and complications*

Continuous weekdays i.p. exchanges of 5-FU 200 mg m$^{-2}$ day$^{-1}$ lasting i.p. to 3 months in one patient and for a shorter time for the other patients resulted in no treatment-associated toxicity worse than WHO grade 2. Those WHO grade 2 toxicity incidences included nausea and vomiting and diarrhoea occurring in two patients after 6 weeks of therapy and was treated successfully with antiemetics and loperamide as an antidiarrhoeal agent. During the whole 235 fluid exchanges or 470 connections and disconnections, no bacterial peritonitis or exit site infection events were observed.

### Discussion

The study clearly demonstrates the feasibility of long-term continuous weekdays administration of 5-FU i.p. chemotherapy for up to 3 months on an ambulatory treatment basis. This report once again demonstrates the pharmacological advantage that can be achieved by i.p. instillation of chemotherapy. 5-FU concentration ratios between the peritoneal fluid and plasma were about 1,000 for all patients. Interestingly, the peritoneal and plasma 5-FU concentration versus time semilogarithmic curves are parallel for up to 12 h post i.p. instillation, leading to an apparent plasma 5-FU half-life similar to the i.p. 5-FU half-life of around 1.3 h, which is significantly longer than plasma 5-FU half-life of 0.2 h when 5-FU was administered by the i.v. route (Goldberg *et al.*, 1988). Furthermore, the novel carrier solution did not affect the 5-FU pharmacokinetic parameters compared with previous studies with other carrier solutions (1.5% dextrose dialysate or lactated Ringer's solution) (Speyer *et al.*, 1980, 1981; Demicheli *et al.*, 1982; Arbuck *et al.*, 1986; Campora *et al.*, 1987; Schilsky *et al.*, 1990; Sugarbaker *et al.*, 1990).

The relatively constant peritoneal fluid balance achieved with this new carrier solution ensures maximal coverage of peritoneal areas liable to bear tumours for 24 h. This allows a single daily exchange, which is a much more viable prospect for prolonged out-patient dialysis than some of the schedules previously used (for example eight 4-hourly exchanges repeated monthly) (Speyer *et al.*, 1980, 1981; Arbuck *et al.*, 1986). The constant peritoneal fluid balance achieved with the carrier solution combined with the 'state of the art' peritoneal twin-bag delivery system gives long-term and feasible access to the peritoneal cavity with no incidence of exit site infection and bacterial peritonitis in this feasibility study.

Based on the pharmacokinetic parameters from the present study we have designed a loading dose infusion schedule in an attempt to maintain steady-state i.p. 5-FU levels.

We have initiated a multicentre phase I study in collaboration with ML Laboratories to determine the maximum tolerable dose (MTD) values for continuous long-term i.p. treatment for 3 months in patients with advanced colorectal, gastric and ovarian cancer.

The authors gratefully acknowledge the expert support of Dr F. Mutch for the cytological studies, thank R. Blackie for his expert technical assistance in 5-fluorouracil analysis in biological fluids, and are indebted to ML Laboratories PLC for the provision of icodextrin solutions. Professor D.J. Kerr is supported by the Cancer Research Campaign, UK.

### References

ANTONSEN, S., PEDERSON, F.B., WANG, P. & THE DANISH STUDY GROUP ON PERITONITIS DIALYSIS (DASPID) (1991). Leukocytes in peritoneal dialysis effluents. *Perit. Dial Int.*, **11**, 43-47.

ARBUCK, S.G., TRAVE, F., DOUGLASS, Jr. H.Q., NAVA, H., ZAKZEWSKI, S. & RUSTUM, Y.M. (1986). Phase I and pharmacologic studies of intraperitoneal leucovorin and 5-fluorouracil in patients with advanced cancer. *J. Clin. Oncol.*, **4**, 1510-1517.

BATES, C.D., WATSON, D.G., WILLMOTT, N., LOGAN, H. & GOLDBERG, J. (1991). The analysis of 5-fluorouracil in human plasma by gas chromatography – negative ion chemical ionization mass spectrometry (GG-NICIMS) with stable isotope dilution. *J. Pharmacol. Biomed. Anal.*, **9**, 19-21.

BENGMARK, S. (ed). (1989). *The Peritoneum and Peritoneal Access*, pp. 1-366. Butherworth: Cambridge.

CAMPORA, E., ESPOSITO, M., CIVALLERI, D., GOGIOSO, L., DECIAN, F., BALLETO, N., FALCONE, A., NOBILE, M.T., PARODI, B., CAFAGGI, S. & BIGNARDI, G. (1987). Serum, urine and peritoneal fluid levels of 5-FU following intraperitoneal administration. *Anticancer Res.*, **7**, 829-832.

CASPER, E.S., KENSEN, D.P., ALCOCK, N.W. & LEWIS, J.L. (1983). IP cisplatin in patients with malignant ascites: pharmacokinetic evaluation and comparison with the IV route. *Cancer Treat. Rep.*, **67**, 235-238.

CHRISTOPHIDIS, N., MIHALY, G., VAJDA, F. & LOUIS, W. (1979). Comparison of liquid and gas-liquid chromatographic assays of 5-fluorouracil in plasma. *Clin. Chem.*, **25**, 83-86.

CLARKSON, B., O'CONNOR, A., WINSTON, C. & HUCHISON, D. (1964). The physiologic disposition of 5-fluorouracil and 5-fluoro-2'-deoxyuridine in man. *Clin. Pharmacol. Ther.*, **5**, 581-610.

DEMICHELI, R., JIRILLO, A., BONCIARELLI, G., BELLINI, A., PETROSINO, L., BIGI, L. & GARUSI, G.F. (1982). Pharmacological data and technical feasibility of intraperitoneal 5-fluorouracil administration. *Tumori*, **68**, 437-441.

EKBERG, H., TRANBERG, K.G., PERSSON, B., JEPPSSON, B., NILLSON, L.-G., GUSTAFSON, T., ANDERSSON, K.I. & BENGMARK, S. (1988). Intraperitoneal infusion of 5-FU in liver metastases from colorectal cancer. *J. Surg. Oncol.*, **37**, 94-99.

FOK, F.K., BEWTRA, C. & HAMMEKE, M.D. (1989). Cytology of peritoneal fluid from patients on continuous ambulatory peritoneal dialysis. *Acta Cytol.*, **33(5)**, 595-598.

GOLDBERG, J.A., KERR, D.J., WILLMOTT, M., McKILLOP, J.H. & McARDLE, C.S (1988). Pharmacokinetics and pharmacodynamics of locoregional 5-fluorouracil (5FU) in advanced colorectal liver metastases. *Br. J. Cancer*, **57**, 186-189.

HALLENBECK, P., SANNIEZ, C.K., RYAN, A.B., NEILEY, B. & SUGARBAKER, P.H. (1992). Cytoreductive surgery and intraperitoneal chemotherapy, treatment for peritoneal carcinomatosis. *AORN J.*, **56**, 50-72.

LEICHMAN, C.G., LEICHMAN, L., SPEARS, C.P., ROSEN, P.J., JEFFERS, S. & GROSHEN, S. (1993). Prolonged continuous infusion of fluorouracil with weekly bolus leucovorin: a phase II study in patients with disseminated colorectal cancer. *J. Natl Cancer Inst.*, **85**, 41-44.

LOKICH, J.J., AHLGREN, J.D., GULLO, J.J., PHILIPS, J.A. & FRYER, J.G. (1989). A prospective randomized comparison of continuous infusion fluorouracil with a conventional bolus schedule in metastatic colorectal carcinoma: a Mid-Atlantic Oncology Program study. *J. Clin. Oncol.*, **7**, 425-432.

MISTRY, C.D., MALLICK, N.P. & GOKAL, R. (1985). The advantage of glucose polymer as an osmotic agent in continuous peritoneal dialysis. *Proc. EDTA-ERA*, **22**, 415-420.

MISTRY, C.D., MALLICK, N.P. & GOKAL, R. (1987). Ultrafiltration with an isosmotic solution during long peritoneal dialysis exchanges. *Lancet*, **ii**, 178-182.

PICCART, M.J., SPEYER, J.L., MARKMAN, M., TEN BOKKEL HUININK, W.W., ALBERTS, D., JENKINS, J. & MUGGIA, F. (1985). Intraperitoneal chemotherapy: technical experience at five institutions. *Semin. Oncol.*, **12** No. 3 (Suppl. 4), 90-96.

ROTTEMBOURG, J., BROUARD, R., ISSAD, B., ALLOUACHE, M. & JACOBS, C. (1987). Prospective randomized study about Y-connectors in CAPD patients. In *Advances in Continuous Ambulatory Peritoneal Dialysis*. Khonna, R., Norph, K.D., Prowant, B., Twardowski, Z.J. & Oreopoulos, D.G. (eds) pp. 107-113. Toronto Peritoneal Dialysis Bulletin: Toronto.

SCHILSKY, R.L., CHOI, K.E., GRAYHACK, J., GRIMMER, D., GUARNIERI, C. & FULLEM, L. (1990). Phase I clinical and pharmacological study of intraperitoneal cisplatin and fluorouracil in patients with advanced intra-abdominal cancer. *J. Clin. Oncol.*, **8**, 2054-2061.

SEIFERT, P., BAKER, L.H., REED, M.L. & VAITKEVICIUS, (1975). Comparison of continuously infused 5-fluorouracil with bolus injection in treatment of patients with colorectal adenocarcinoma. *Cancer*, **36**, 123-128.

SPEYER, J.L., COLLINS, J.M., DEDRICK, R.L., BRENNAN, M.F., BUCKPITT, A.R., LONDER, H., DE VITA, V.T. & MYERS, CH.E. (1980). Phase I and pharmacological studies of 5-fluorouracil administered intraperitoneally. *Cancer Res.*, **40**, 567-572.

SPEYER, J.L., SUGARBAKER, P.H., COLLINS, J.M., DEDRICK, R.L., KLECKER, Jr. R.W. & MYERS, CH.E. (1981). Portal levels and hepatic clearance of 5-fluorouracil after intraperitoneal administration in humans. *Cancer Research*, **41**, 1916-1922.

SPEYER, J.L. (1985). The rationale behind intraperitoneal chemotherapy in gastrointestinal malignancies. *Semin. Oncol.*, **12** No. 3 (Suppl. 4), 23-28.

SUGARBAKER, P.H., GIANOLA, F.J., SPEYER, F.C., WESLEY, R., BAROFSKY, I. & MEYERS, CH.E. (1985). Prospective, randomized trial of intravenous versus intraperitoneal 5-fluorouracil in patients with advanced primary colon or rectal cancer. *Surgery*, **98**, 414-421.

SUGARBAKER, P.H., GRAVES, T., DE BRUIJN, E.A., CUNLIFFE, W.J., MULLIN, R.E., HULL, W.E., OLIFF, L. & SCHLAG, P. (1990). Early postoperative intraperitoneal chemotherapy as an adjuvant therapy for surgery for peritoneal carcinomastosis from gastrointestinal cancer: pharmacological studies. *Cancer Res.*, **50**, 5790-5795.

SUGARBAKER, P.H. (1991). Mechanisms of relapse for colorectal cancer: Implications for Intraperitoneal Chemotherapy. *J. Surg. Oncol.*, (Suppl. 2), 36-41.

SUHRLAND, L.G. & WEISBERGER, A.S. (1965). Intracavity 5-fluorouracil in malignant effusions. *Arch. Int. Med.*, **116**, 431-433.

VERGER, C., LUZAR, M.A. (1986). In vitro study of the CAPD Y-line systems. In *Advances in Continuous Ambulatory Peritoneal Dialysis*, Khonna, R., Norph, K.D., Prowant, B., Twardowski, Z.J. & Oreopoulos, D.G. (eds) pp. 160-164. Toronto University Press: Toronto.

WAHL, R.L., GYVES, J., GRASS, B.H., COCKRAN, M., JUNI, J.E., ARNSTEIN, N.B., LAHTI, D. & ACKERMANN, R.J. (1989). SPECT of the peritoneal cavity: method for delineating intraperitoneal fluid distribution. *Am. J. Reontgenol.*, **152**, 1205-1210.

WEISBERGER, A.S., LEVINE, B. & STORAASLI, J.P. (1955). Use of nitrogen mustard in treatment of serous effusions of neoplastic origin. *JAMA*, **12**, 1704-1707.